# Functional and Structural Long-Term Effects of Repetitive Instrument-Assisted Manual Therapy (IAMT) of the Lumbar Back on the Dorsal Myofascial Chain Among Female Soccer Players: A Randomized, Placebo-Controlled Pilot Trial

**DOI:** 10.7759/cureus.69337

**Published:** 2024-09-13

**Authors:** Patrick Weber, Werner Klingler, Robert Schleip, Nadine Weber, Christine Joisten

**Affiliations:** 1 Department of Physical Activity in Public Health, Institute of Movement and Neurosciences, German Sport University Cologne, Cologne, DEU; 2 Department of Physiotherapy, Medical Cooperative Society, PANOVIA, Hürth, DEU; 3 Department of Anaesthesiology, SRH Hospital Sigmaringen, Sigmaringen, DEU; 4 Department of Experimental Anaesthesiology, Ulm University, Ulm, DEU; 5 Department of Clinical Sciences, Queensland University of Technology, Brisbane, AUS; 6 Department of Sport and Health Sciences, Technical University of Munich, Munich, DEU; 7 Department of Medical Professions, Diploma University of Applied Sciences, Bad Sooden-Allendorf, DEU; 8 Department of Medicine, Medical Cooperative Society, PANOVIA, Hürth, DEU

**Keywords:** hamstring injury, instrument-assisted, lumbar fascia, manual therapy, ultrasonography

## Abstract

Introduction: Instrument-assisted manual therapy (IAMT) is suitable for optimizing movement prerequisites, such as range of motion, flexibility, microcirculation, and pain inhibition along myofascial chains, potentially leading to a reduction in hamstring injuries. To date, however, IAMT’s modes of action remain largely unclear. This study aimed to examine the functional and structural effects of repetitive IAMT after 2.5 and five weeks.

Methods: Sixteen healthy female soccer players (age: 21.4 (±5.1) years) were randomly grouped into an intervention group and a placebo control group. The intervention group received nine IAMT sessions twice weekly at the right lumbar region. The placebo group received a single pressureless “therapy” at baseline. In addition to structural ultrasound analyses (absolute motion and shear motion), functional mobility tests (passive straight leg raise (PSLR) test and lumbar and thoracic double inclinometry) were performed 4.0 (±2.0) days after the fourth IAMT and 3.4 (±1.1) days after the ninth IAMT in both groups.

Results: Hamstring flexibility in the PSLR test improved significantly after the ninth IAMT compared with the placebo group (*p* < 0.05, effect size: 0.913). No systematic differences were seen at the structural level.

Conclusion: Repetitive IAMT can improve hamstring flexibility. Further studies in larger groups and diverse collectives are necessary to additionally test the postulated preventive effect also on hamstring injuries. Whether ultrasound is the right method for detecting structural changes in this context needs to be verified in the future.

## Introduction

In sports, hamstring injuries cause long periods of absence and fall into the category of serious injuries. In soccer, these injuries are at the top of the list of the most common injuries, meaning that sustained preventive measures are urgently needed [1,2]. In addition to physical approaches in the form of strength training, the functionality of myofascial chains is increasingly coming into focus [3-7].

In the aforementioned context, instrument-assisted manual therapy (IAMT) has been used to optimize movement prerequisites alongside actively applied myofascial self-release exercises, such as foam rolling, potentially leading to a reduction in hamstring injuries [5,6,8-12]. Even a single two-minute IAMT session on the front and back thighs has been observed to result in better and more sustained range of motion (ROM) adaptations compared with foam rolling of identical location and time in soccer players [6]. Furthermore, in patients with non-specific low back pain, a single intervention on the back of the thigh has improved hamstring flexibility and reduced lower back pain [13]. Consequently, based on the myofascial connection between the hamstring muscles and the lower back, a reverse intervention effect, as already demonstrated due to fascial stretching, can be hypothesized for IAMT of the lumbar back [14-17].

Despite the abovementioned and complementary findings from other body regions, IAMT’s mechanisms of action remain largely unclear [8,18,19]. In a previous study, we demonstrated that immediately after a single IAMT session on the right lumbar region of healthy female soccer players, there was a significant improvement in hamstring flexibility compared with that after a pressureless placebo intervention [20]. Ultrasound analysis was able to detect previously undetected changes at the structural level: a short-term decrease in the absolute mobility of the superficial lamina of the thoracolumbar fascia and a reduction in the shear motion (SM) relative to the overlying superficial fascia. Forty-five minutes after IAMT, structural adjustments normalized, with the absolute mobility of the superficial lamina of the thoracolumbar fascia increasing above its baseline level. Contrary to this opposing structural development, hamstring flexibility showed sustained and further improvements over time [20].

Based on the aforementioned background, we analyzed the five-week long-term data of our pilot study to gain more detailed insights into the effects of standardized IAMT of the lumbar back on the functional and structural properties of the dorsal myofascial chain in healthy female soccer players: a homogeneous and available high-level collective. As in the short-term effects, we aimed to analyze the structural movement outcomes, including the absolute movement of the different tissue layers and the relative movement between two neighboring tissue layers defined as the SM (primary outcome), and the functional movement outcomes, such as the flexibility of the hamstring muscles (primary outcome) and the lumbar and thoracic spines (secondary outcome). Thereby, the focus was on verifying our initial findings already published on the effects of a single IAMT session, also over a period of repetitive IAMT sessions, and quantifying the expected adaptations at the structural level. In this context, we hypothesized that our functional movement parameters along the dorsal myofascial chain would improve, leading to a greater ROM, as a result of repetitive IAMT of the lumbar back. This is particularly expected locally in the intervention area and especially due to the hypothesized inverse interaction between the lower back and posterior thigh in hamstring flexibility. Furthermore, an improvement in our structural movement parameters was assumed, which is expressed in greater absolute mobility of the individual tissue layers and leads to an improved SM between the adjacent tissue layers, especially in the thoracolumbar fascia.

## Materials and methods

Study design

**Figure 1:**
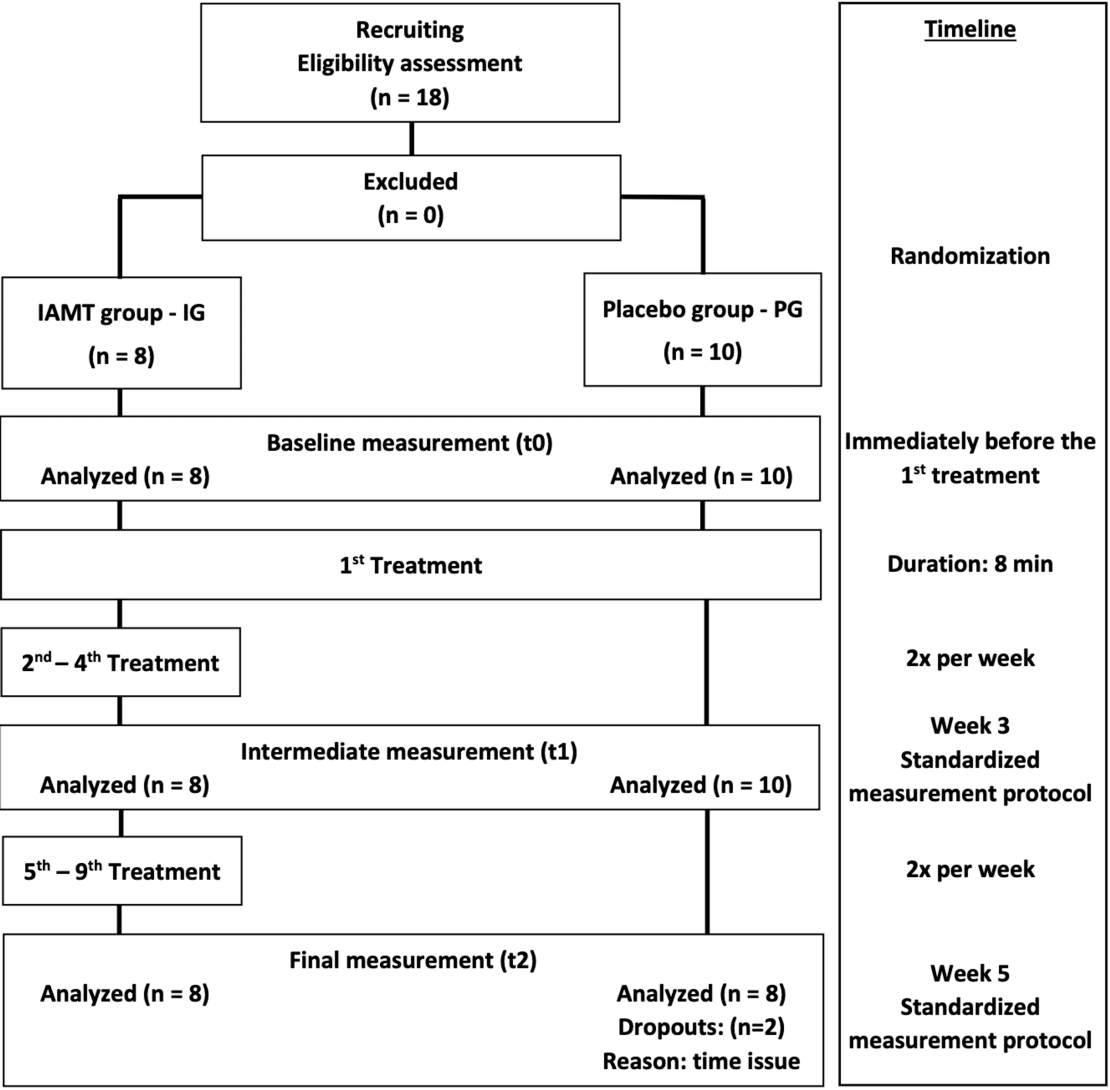
Flow Chart of the Long-Term Part of This Pilot Study IAMT: instrument-assisted manual therapy; IG: intervention group; PG: placebo group.

This long-term evaluation was a pilot part of a randomized, placebo-controlled, and blinded study and followed the standardized methodological procedure [21]. The study was registered at the German Clinical Trials Register (DRKS00012252) and received ethical approval from the ethics committee of the German Sport University Cologne (No. 80/2017) in accordance with the latest version of the Declaration of Helsinki. For general group allocation, the soccer players were randomized into three groups using equal-sized printed cards (1:1:1) with group assignments in sealed envelopes (urn design) [21-23]. After the short-term analysis (comparison of all three groups), the study immediately moved on to the long-term analysis (only two groups) with the participants of the intervention group (IG) and placebo-control group (PG) who had agreed voluntarily to participate (Appendix Figure 3). In the long-term evaluation between these two groups, the IG received nine standardized IAMT sessions twice weekly over five weeks with a minimum of two days between two consecutive sessions. The PG received a pressureless placebo treatment once on the right side of the lower back at baseline and continuously served as the classical control group. The left side remained untreated and served as a control side. Measurements were performed at baseline (t0), after 15.8 (±2.0) days (t1), and 4.0 (±2.0) days (minimum two days and maximum seven days) after the fourth session but immediately before the fifth session. After five weeks (34.1 [±2.2] days) and 3.4 (±1.1) days (minimum two days and maximum five days) after the ninth IAMT session, the final measurements (t2) were conducted for both groups. At that time, the possible placebo treatment effects should have already subsided (Figure 1). A follow-up measurement was performed 8-12 days after t2. Since this measurement was performed for the IG only, it was not included in the present analysis.

Participants

Eighteen healthy female soccer players aged 15-35 years enrolled from October 2017 to March 2018 in the short-term part of this pilot study agreed to participate in the long-term investigation (Appendix Table 6) [20]. All participants (and, in the case of minors, additionally the parents) signed informed consent forms after receiving detailed verbal and written information about the study.

Measurements

Anthropometric data were recorded, and a specific questionnaire about the athletes’ career, lifestyle, and practice sessions was analyzed. The standardized measurement protocol was applied reciprocally on both sides of the body [21]. Ultrasound imaging was followed by flexibility measurements, starting with the intervention side each time, to minimize potential effects due to the measurement sequence, which were therefore not expected and were not pursued further. The room temperature was maintained from 22.9 (±0.4)°C to 24.4 (±0.3)°C in both groups throughout the study protocol and there were no differences in the required examination time and the time interval between the measurements.

Ultrasound Imaging

Ultrasound imaging was performed to evaluate the absolute movement and SM of the lower back’s different tissue layers (SonidoSmart Plus, Zimmer MedizinSysteme, Neu-Ulm, Germany) as previously described by Weber et al. [20]. Therefore, the athletes lay in a prone position with their anterior superior iliac spine above the rotation axis of the treatment table. For the imaging, a linear transducer (16 MHz) with a length of 3.8 cm was placed longitudinally 2 cm lateral to the spinous processes of the lumbar vertebrae (L) 2 and L3. A single ultrasound beam was focused on the thoracolumbar fascia at its posterior layer with a depth of 3.2 cm. For the motion analysis of the tissue layers, a cine-loop technique recorded the required video sequences during the treatment table was electrically tilted at a speed of 3° per second to a roof position of 45°. The absolute mobility and SM were then analyzed with the cross-correlation software “Motion Analysis 2014v1” [20,21].

Dorsal Structure Flexibility Tests

The passive straight leg raise (PSLR) test was performed in a rested supine position with extended legs. The tested leg was raised to maximum passive hip flexion until a noticeable resistance or palpable pelvic rotation occurred. Hamstring flexibility was determined in degrees using an inclinometer (AcuAngle, Baseline, Elmsford, New York, USA) centered at the level of the lateral malleolus [20,24,25].

For lumbar and thoracic spine flexibility, double inclinometry was used: Based on the standardized protocol, the athletes were guided to bend over from an upright position with their legs extended, first passively by gravity and then by muscle activation [20]. For lumbar spine flexibility, the upper inclinometer was centered on the motion segment of the thoracic vertebra (Th) 12 and L1 while the lower one was placed at the sacral base. For thoracic spine flexibility the inclinometer at the motion segment of Th12 and L1 was held in position and the sacral one moved upwards over the motion segment of Th1 an Th2 using the same test protocol. The respective flexibility was calculated based on the difference in the upper and lower inclinometers in degrees [26-29].

Treatments

Both groups were treated in the prone 45° roof position at the defined intervention area, which extended from the spinous processes between the 12th rib and the iliac crest to the connecting line of the costal arch and iliac crest on the right side of the body (Figure 4) [20].

IAMT Treatment

The IAMT was conducted standardized per the intervention protocol by Weber et al., which started with the metabolization and followed by the rehydration technique [20]. During metabolization, short frontal shock-like frictions with the convex side of the intervention instrument (Fazer 2, Ludwig Artzt GmbH, Dornburg, Germany) were applied to the tissue in the direction of movement. During rehydration, the intervention instrument was slowly slid with the convex side through the tissue, shifting a skin fold in front of the instrument. The two techniques were applied at the pressure-pain threshold (defined as the maximum pressure before it turns into discomfort) described by Rolke et al., per instructions by Weber et al. using a minimal sliding layer of pH-neutral cream [20,30]. Each technique consisted of three overlapping lines in six different directions to reach as many fiber courses as possible (Appendix Figure 4) [20]. After the three lines were completed twice, the subsequent direction was continued. The applied average pressure of all treatment directions reached for metabolization 6.8 (±1.2) kg at an average speed of 6.3 (±0.5) cm/s and for rehydration 8.0 (±0.9) kg at a speed of 2.1 (±0.3) cm/s.

Placebo Treatment

The placebo treatment was performed using an inactivated ultrasound transducer with a surface area of 5 cm^2^. With a conductive gel applied, the transducer was moved circularly at a moderate pace and without added pressure over a duration of 8 min.

Statistical analysis

All statistical analyses were conducted using IBM SPSS Statistics for Windows, Version 29 (Released 2023; IBM Corp., Armonk, New York, United States). The results were summarized and presented using descriptive statistics. For all measurement time points, all quantitative group characteristics, and main outcomes, such as absolute mobility, SM, and flexibility, were compared using an unpaired t-test or the Mann-Whitney U test in the absence of normal data distribution determined by the Kolmogorov-Smirnov-Test. Based on the current literature, a one-sided t-test was indicated and used for the functional measurement parameters, and a two-sided t-test for all other measurement parameters [9]. For the intermediate mean comparison, delta (Δ1) values were calculated using the intermediate measurement (t1) and baseline measurement (t0) data. For the final mean comparison, delta (Δ2) values were calculated using the final measurement (t2) and baseline measurement (t0) data. Qualitative variables, such as performance level and myofascial self-release experience, were evaluated using the chi-squared test. The effect size was calculated based on Cohen’s d for the unpaired t-test parameters and on the correlation coefficient (r) for the Mann-Whitney U test parameters. The significance level was set at p < 0.05.

## Results

Of the 18 female soccer players included in the long-term part of this pilot study, 16 participated in the final measurements. Two athletes in the PG did not participate in the final measurements owing to time constraints. At baseline, the average age of the 16 players was 21.4 (±5.1) years with a mean height of 167.4 (±5.6) cm, and a mean weight of 62.1 (±8.3) kg. Both groups had no differences in any quantitative and qualitative characteristics at all measurement time points (Table 1).

**Table 1:** Quantitative and Qualitative Group Characteristics Quantitative group characteristics are presented as means ± SDs and qualitative group characteristics as numbers with p-values. Menstruation t0: All: n = 15, IG: n = 7, PG: n = 8. Test: †unpaired t-test, ‡Mann–Whitney U test, #chi-squared test. IG: intervention group (instrument assisted manual therapy treatment); PG: placebo group (pressurless placebo treatment); SD: standard deviation; BMI: body mass index; MSR: myofascial self-release experience.

Parameter		All (n = 16)		IG (n = 8)		PG (n = 8)		
		mean	±SD	mean	±SD	mean	±SD	p-value
Baseline (t0)								
Age (year)		21.4	±5.1	20.4	±4.8	22.3	±5.6	0.279^‡^
Height (cm)		167.4	±5.6	167.4	±4.7	167.5	±6.7	0.966^†^
Weight (kg)		62.1	±8.3	63.0	±9.1	61.2	±7.9	0.666^†^
BMI (kg/m²)		22.2	±2.7	22.5	±2.9	21.8	±2.7	0.878^‡^
Sport experience (year)		12.4	±5.5	10.9	±4.7	13.9	±6.2	0.328^‡^
Weekly extent (hour)		7.9	±1.8	7.7	±1.6	8.2	±2.1	0.557^†^
7-day-extent (hour)		5.8	±2.9	6.0	±2.7	5.5	±3.3	0.764^†^
Menstruation (day)		17.1	±8.4	18.4	±9.8	15.9	±7.4	0.575^†^
Intermediate (t1)								
Weight (kg)		62.1	±8.4	63.3	±9.1	61.0	±8.1	0.442^‡^
BMI (kg/m²)		22.2	±2.8	22.6	±2.9	21.8	±2.8	0.442^‡^
7-day-extent (hour)		3.9	±2.7	2.7	±1.7	5.0	±3.2	0.094^†^
Menstruation (day)		18.2	±8.3	14.8	±7.4	21.6	±8.2	0.100^†^
Final (t2)								
Weight (kg)		62.0	±8.4	62.8	±9.2	61.2	±8.0	0.721^‡^
BMI (kg/m²)		22.1	±2.7	22.4	±2.9	21.8	±2.7	0.721^‡^
7-day-extent (hour)		5.5	±2.2	5.1	±2.7	5.9	±1.7	0.511^†^
Menstruation (day)		12.4	±7.7	12.3	±8.8	12.6	±7.0	0.926^†^
		n		n		n		p-value
Baseline (t0)								
Level	State	3		1		2		-
-	National	11		6		5		0.809^#^
-	International	2		1		1		-
Squad	None	11		5		6		-
-	State	2		2		0		0.298^#^
-	National	3		1		2		-
Playing leg	Right	11		5		6		0.590^#^
-	Left	5		3		2		-
MSR	Yes	12		6		6		1.000^#^
-	No	4		2		2		-
Hormonal	Yes	7		3		4		0.614^#^
contraception	No	9		5		4		-

Structural movement parameters

The analysis of the structural movement parameters showed greater absolute mobility of the left erector spinae muscle at baseline in the IG than in the PG (p < 0.05; Table 2). The intermediate and final measurements revealed no differences between both groups (Table 3).

**Table 2:** Structural Movement Parameters (Mean Values) Absolute movement and shear motion are shown as means ± SDs. *p < 0.05. Test: †unpaired t-test, ‡Mann–Whitney U test. Baseline (t0): baseline measurement immediately before the first intervention; Intermediate (t1): intermediate measurement immediately before the fifth intervention; Final (t2): final measurement five weeks after the first intervention; IG: intervention group; PG: placebo group; SD: standard deviation; SF: superficial fascia; TLF: thoracolumbar fascia; SL: superficial lamina; DL: deep lamina; ESM: erector spinae muscle; R: right; L: left; SM: shear motion.

Parameter	Baseline (t0)					Intermediate (t1)				Final (t2)			
	IG (n = 8)		PG (n = 8)			IG (n = 8)		PG (n = 8)		IG (n = 8)		PG (n = 8)	
	mean	±SD	mean	±SD	p-value	mean	±SD	mean	±SD	mean	±SD	mean	±SD
SF R (mm)	3.41	±2.53	3.73	±2.38	0.800^†^	3.26	±1.66	3.73	±1.83	4.02	±1.87	3.07	±1.86
TLF-SL R (mm)	5.96	±3.98	4.84	±3.49	0.558^†^	6.13	±3.95	5.16	±2.69	6.14	±3.03	4.54	±2.94
TLF-DL R (mm)	9.81	±4.14	9.40	±5.09	0.864^†^	10.86	±5.46	9.53	±3.29	11.47	±3.61	8.90	±2.81
ESM R (mm)	14.47	±2.58	13.20	±4.55	0.505^†^	15.84	±4.58	12.72	±3.98	15.37	±4.58	12.31	±2.26
SF L (mm)	2.76	±1.91	3.37	±1.56	0.495^†^	4.07	±1.20	3.50	±1.17	4.10	±1.44	3.68	±1.36
TLF-SL L (mm)	6.14	±3.69	5.21	±1.92	0.537^†^	7.18	±2.71	5.83	±2.91	7.70	±3.62	6.11	±2.65
TLF-DL L (mm)	9.29	±3.15	9.22	±2.20	0.798^‡^	11.32	±2.81	9.45	±3.28	12.05	±2.74	9.38	±3.77
ESM L (mm)	14.04	±2.38	11.32	±2.63	0.048^†*^	14.03	±3.45	11.41	±1.99	14.00	±3.80	11.39	±2.44
SM (SF/TLF-SL) R (mm)	2.55	±1.92	1.11	±1.26	0.101^†^	2.87	±2.50	1.44	±1.22	2.12	±1.54	1.47	±1.26
SM (TLF-SL/TLF-DL) R (mm)	3.85	±1.99	4.57	±3.25	0.878^‡^	4.73	±2.32	4.36	±2.23	5.33	±1.95	4.36	±1.59
SM (TLF-DL/ESM) R (mm)	4.66	±3.94	3.80	±4.03	0.672^†^	4.97	±2.99	3.19	±4.17	3.90	±2.71	3.41	±1.76
SM (SF/TLF-SL) L (mm)	3.39	±2.37	1.85	±0.73	0.115^†^	3.12	±1.95	2.33	±2.01	3.61	±3.15	2.43	±1.80
SM (TLF-SL/TLF-DL) L (mm)	3.14	±1.52	4.01	±2.24	0.380^†^	4.14	±2.95	3.61	±1.66	4.35	±2.59	3.27	±1.61
SM (TLF-DL/ESM) L (mm)	4.76	±3.32	2.10	±2.80	0.105^†^	2.71	±3.87	1.97	±2.26	1.95	±2.94	2.01	±2.91

**Table 3:** Structural Movement Parameters (Delta Values) Absolute movement and shear motion are shown as Δ1 (t1 − t0) and Δ2 (t2 − t0) ± SDs with p-values and effect sizes. *p < 0.05. Effect size: Cohen’s d. Test: †unpaired t-test, ‡Mann–Whitney U test. Intermediate (t1): intermediate measurement immediately before the fifth intervention; Final (t2): final measurement five weeks after the first intervention; IG: intervention group; PG: placebo group; SD: standard deviation; SF: superficial fascia; TLF: thoracolumbar fascia; SL: superficial lamina; DL: deep lamina; ESM: erector spinae muscle; R: right; L: left; SM: shear motion.

Parameter	Intermediate (t1)					Final (t2)				
	IG (n = 8)		PG (n = 8)			IG (n = 8)		PG (n = 8)		
	Δ1	±SD	Δ1	±SD	p-value	Δ2	±SD	Δ2	±SD	p-value
SF R (mm)	−0.15	±2.32	−0.01	±1.89	0.893^†^	0.60	±2.22	−0.66	±2.20	0.328^‡^
TLF-SL R (mm)	0.17	±2.65	0.33	±2.69	0.908^†^	0.18	±2.84	−0.30	±3.18	0.721^‡^
TLF-DL R (mm)	1.05	±4.80	0.12	±3.26	0.442^‡^	1.66	±4.19	−0.50	±4.97	0.362^†^
ESM R (mm)	1.37	±3.75	−0.49	±5.06	0.418^†^	0.90	±3.26	−0.90	±4.39	0.368^†^
SF L (mm)	1.31	±1.31	0.14	±1.48	0.115^†^	1.34	±2.06	0.31	±1.45	0.267^†^
TLF-SL L (mm)	1.04	±1.83	0.62	±2.69	0.721^†^	1.56	±2.78	0.90	±2.91	0.650^†^
TLF-DL L (mm)	2.04	±2.91	0.23	±2.36	0.193^†^	2.77	±2.72	0.16	±2.19	0.083^‡^
ESM L (mm)	−0.01	±3.04	0.09	±2.54	0.943^†^	−0.05	±2.68	0.07	±2.63	0.932^†^
SM (SF/TLF-SL) R (mm)	0.32	±1.07	0.33	±1.16	0.981^†^	−0.42	±1.00	0.36	±1.09	0.156^†^
SM (TLF-SL/TLF-DL) R (mm)	0.88	±3.06	−0.21	±2.67	0.461^†^	1.48	±2.33	−0.21	±3.26	0.254^†^
SM (TLF-DL/ESM) R (mm)	0.31	±2.56	−0.61	±3.74	0.573^†^	−0.76	±2.29	−0.39	±3.58	0.811^†^
SM (SF/TLF-SL) L (mm)	−0.27	±0.82	0.48	±1.71	0.382^‡^	0.22	±1.49	0.59	±1.63	0.643^†^
SM (TLF-SL/TLF-DL) L (mm)	1.00	±2.86	−0.39	±1.47	0.242^†^	1.21	±2.56	−0.74	±1.21	0.073^†^
SM (TLF-DL/ESM) L (mm)	−2.05	±3.95	−0.14	±2.27	0.255^†^	−2.81	±2.99	−0.09	±2.00	0.050^†^

Functional movement parameters

**Figure 2:**
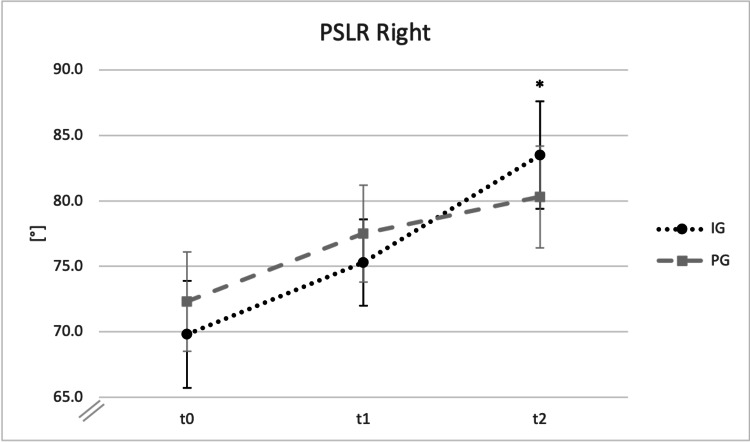
Passive Straight Leg Raise Test Results of the Right Leg in Degrees. Means ± SEs for the Baseline, Intermediate, and Final Measurements Are Shown Baseline (t0): baseline measurement immediately before the first intervention; Intermediate (t1): intermediate measurement immediately before the fifth intervention; Final (t2): final measurement five weeks after the first intervention; *p < 0.05. PSLR: passive straight leg raise test; IG: intervention group; PG: placebo group.

**Figure 4:**
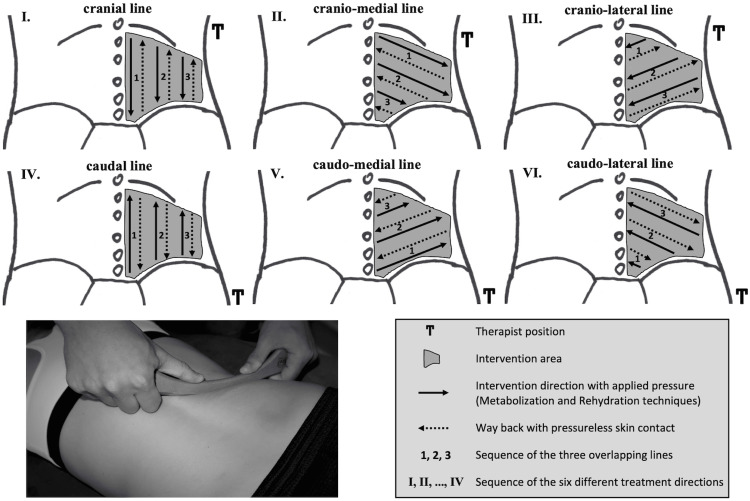
Illustration of the chronological treatment sequences of the instrument-assisted manual therapy. The photograph shows the instrument-assisted manual therapy with the intervention tool Fazer 2 from ARTZT vitality (Ludwig Artzt GmbH, Dornburg, Germany) on the cranio-lateral line Adapted from the studies by Weber et al. [20,21].

There were no significant differences in the functional movement parameters between both groups at baseline (Table 4). A reduced active thoracic spine flexibility was noted in the IG compared with that in the PG in the intermediate measurement (p < 0.05) with an effect size of d = -1.559 (Table 5). In the final measurement, the PSLR test showed an increased ROM on the intervention side in the IG compared with that in the PG (p < 0.05) with an effect size of d = 0.913 (Table 5 and Figure 2).

**Table 4:** Functional Movement Parameters (Mean Values) The passive straight leg raise test and double inclinometry results are presented as means ± SDs. Test: †unpaired t-test. Baseline (t0): baseline measurement immediately before the first intervention; Intermediate (t1): intermediate measurement immediately before the fifth intervention; Final (t2): final measurement five weeks after the first intervention; IG: intervention group; PG: placebo group; SD: standard deviation; PSLR: passive straight leg raise test; PLS: passive lumbar spine flexibility; ALS: active lumbar spine flexibility; PTS: passive thoracic spine flexibility; ATS: active thoracic spine flexibility; R: right; L: left.

Parameter	Baseline (t0)					Intermediate (t1)				Final (t2)			
	IG (n = 8)		PG (n = 8)			IG (n = 8)		PG (n = 8)		IG (n = 8)		PG (n = 8)	
	mean	±SD	mean	±SD	p-value	mean	±SD	mean	±SD	mean	±SD	mean	±SD
PSLR R [°]	69.8	±11.5	72.3	±10.9	0.331^†^	75.3	±9.3	77.5	±10.4	83.5	±11.7	80.3	±11.0
PSLR L [°]	66.8	±12.7	72.0	±12.7	0.211^†^	75.8	±9.4	77.3	±11.8	80.3	±11.2	80.0	±9.4
PLS [°]	52.3	±10.4	53.3	±12.9	0.433^†^	54.0	±6.7	51.5	±6.0	55.5	±8.9	53.0	±8.8
ALS [°]	53.8	±10.5	55.0	±11.7	0.412^†^	57.5	±8.7	54.3	±6.5	55.5	±8.0	52.8	±8.5
PTS [°]	28.5	±12.6	30.0	±4.3	0.378^†^	24.3	±12.7	31.8	±11.4	28.5	±11.8	33.0	±8.1
ATS [°]	30.8	±13.2	27.3	±9.6	0.277^†^	23.0	±10.5	34.0	±8.8	29.5	±13.1	33.3	±6.2

**Table 5:** Functional Movement Parameters (Delta Values) The passive straight leg raise test and double inclinometry results are presented as Δ1 (t1 − t0) and Δ2 (t2 − t0) ± SDs with p-values and effect sizes. *p < 0.05. Effect size: Cohen’s d. Test: †unpaired t-test, ‡Mann–Whitney U test. Intermediate (t1): intermediate measurement immediately before the fifth intervention; Final (t2): final measurement five weeks after the first intervention; IG: intervention group; PG: placebo group; SD: standard deviation; PSLR: passive straight leg raise test; PLS: passive lumbar spine flexibility; ALS: active lumbar spine flexibility; PTS: passive thoracic spine flexibility; ATS: active thoracic spine flexibility; R: right; L: left.

Parameter	Intermediate (t1)						Final (t2)					
	IG (n = 8)		PG (n = 8)				IG (n = 8)		PG (n = 8)			
	Δ1	±SD	Δ1	±SD	p-value	Effect size	Δ2	±SD	Δ2	±SD	p-value	Effect size
PSLR R [°]	5.5	±4.1	5.3	±6.0	0.462^†^	-	13.8	±4.1	8.0	±7.9	0.045^†*^	0.913
PSLR L [°]	9.0	±5.5	5.3	±7.1	0.128^†^	-	13.5	±5.2	8.0	±9.8	0.105^‡^	-
PLS [°]	1.8	±9.9	−1.8	±10.7	0.254^†^	-	3.3	±7.2	−0.3	±9.5	0.210^†^	-
ALS [°]	3.8	±10.6	−0.8	±9.8	0.196^†^	-	1.8	±8.4	−2.3	±9.0	0.187^†^	-
PTS [°]	−4.3	±8.9	1.8	±9.4	0.105^†^	-	0.0	±12.2	3.0	±8.9	0.279^‡^	-
ATS [°]	−7.8	±11.4	6.8	±6.6	0.004^†*^	−1.559	−1.3	±16.8	6.0	±6.9	0.105^‡^	-

## Discussion

To our knowledge, this study is the first to examine longitudinal data on the structural and functional effects of repetitive IAMT of the lower back involving the dorsal myofascial chain. Previously, measurements such as ROM, functionality, superficial skin temperature, and microcirculation were predominantly obtained immediately after a single intervention [8,13,19,20,31,32].

Herein, repetitive IAMT significantly improved hamstring flexibility even 3-4 days after the ninth session compared with the placebo treatment (effect size: Cohen’s d: 0.913) [33]. This finding further confirms the positive effects of IAMT identified in previous studies regarding functionality and especially ROM [6,8,9,13,18,19]. However, the data in this context are relatively weak. Markovic recorded a sustained (attenuated) improvement in the ROM of the knee and hip joint 24 h after a single two-minute IAMT applied to the hamstring and quadriceps muscles of 19 (±2)-year-old male regional-level soccer players (n = 10) [6]. Blanchette and Normand treated 15 male and female patients aged 47 (±10) years with lateral epicondylitis with two IAMT sessions weekly for five weeks [34]. They found improvements in grip strength, pain perception, and disability one week after the final treatment but without significant difference compared to a control group (n = 12; age = 46 ±10 years) who received standard therapy. In another study, subjective improvements in functionality and pain perception were recorded with the additional use of IAMT (n = 57) versus a solely eccentric strengthening and stretching program (n = 56) over four weeks (two interventions per week) in patients aged 18-65 years with chronic lateral elbow tendinopathy already over a longer period of six and 12 months [18]. These subjective long-term insights are supported by the three-month follow-up observations in the IG in the study by Blanchette and Normand [34].

IAMT’s longer-lasting effects could be attributed to structural adaptations. Manual mobilization has been shown to be successful in preventing postoperative adhesions of abdominopelvic structures in rats [35]. However, in the present study, we did not observe the expected systematic adaptations at the structural level in absolute tissue mobility and SM during the ultrasound examinations, as has been noted in other studies using a single foam roller intervention or comparing healthy patients with chronic back pain patients [7,26]. In the future, the use of a higher-resolution ultrasound transducer and the consideration of further influencing factors of the lateral resolution for more precise visualization and delineation of the filigree and partly low-collagen tissue areas would have to be reconsidered for the investigation of structural adaptations, especially the targeted sliding mobility of the tissue. To what extent structural changes would have been visible by a shorter time interval to the previous intervention or in other unhealthy populations (e.g., patients with back pain) remains a topic for future investigations.

However, other causes may have also led to functional improvements. Our previous analysis of short-term effects showed an increase in superficial skin temperature and hyperemia, both of which may have also influenced the functional properties of the dorsal myofascial chain over a longer term [20]. In addition, a sustained local and segmental tissue tension reduction would have been possible through repetitive mechanical stimulation of local receptors [36,37]. Corresponding beneficial effects have already been documented with chronic headaches, myofascial pain syndrome, systemic inflammatory responses, and post-sport mobility [38-41]. Whether and to what extent the recurrent mechanical stimulation and the resulting thermal stimulus have a lasting effect on viscosity and thus on the sliding properties of the tissue are currently unclear, but the possibility cannot be ruled out [42,43].

The recorded reduction in active thoracic spine flexibility in the intermediate measurement does not correspond to the expected and previously researched findings [6,8,9,13,18,19]. Despite further IAMT applications, this result was not confirmed in the final measurement and returned to the baseline level. This deviating behavior of the active thoracic spine flexibility is inexplicable to us and should be further observed in future studies.

Strengths and limitations

In addition to the study design being randomized, controlled, and blinded, this pilot study is characterized primarily by its standardized examination method and treatment protocol. For the first time, the functional and structural effects of repetitive IAMT were recorded several days after the previous intervention. To the authors' best knowledge, the accurately selected multiplicity of analyses to supplement the functional movement parameters commonly used to date does not appear to have negative influences on the research results. Still, a more detailed examination of the microcirculation, for example, using the Oxygen to See device (LEA Medizintechnik, Heuchelheim, Germany), is recommended in future research, in order to assess possible acute effects and the sustainability of these effects through IAMT [44,45]. In addition, future studies should examine whether the more frequent personal contact with the practitioner could have led to a placebo effect in the IG. Furthermore, only the total weekly training volume was recorded in the present study. In addition, monitoring of the specific training content should be considered in order to control possible influencing factors on the measurement parameters. The extent to which this can be implemented with larger groups and consequently several training groups must be examined individually. In general, larger samples and additional populations with preexisting structural and recurrent impairments of the dorsal myofascial chain should be examined in predefined time series, enabling more precise statements about possible adaptive responses and the timing and duration of the effects. 

## Conclusions

Repetitive IAMT of the lumbar back (two interventions per week) yields a sustained improvement in hamstring flexibility detectable over several days after nine sessions but without evidence of structural adjustments during ultrasound examination. However, the concrete mechanisms behind the treatment-specific effects of IAMT and the duration of these effects remain unclear. It was also found that more than four sessions were needed to achieve sustained adaptation a few days after the previous treatment. Future studies should investigate these mechanisms further, considering mechanical, sensory, and metabolic effects in larger samples. Long-term studies with a larger sample size are necessary to verify the existing results and investigate the currently unverified structural measurement parameters. Additional studies on male soccer players are recommended to transfer the existing knowledge to the other gender in the same sport in order to compare gender-specific differences. Future studies should also be carried out in other sports. Furthermore, athletes with recurrent hamstring injuries should be focused in the future to investigate the initial assumption of hamstring injury prevention by IAMT.

## Figures and Tables

**Figure 3:**
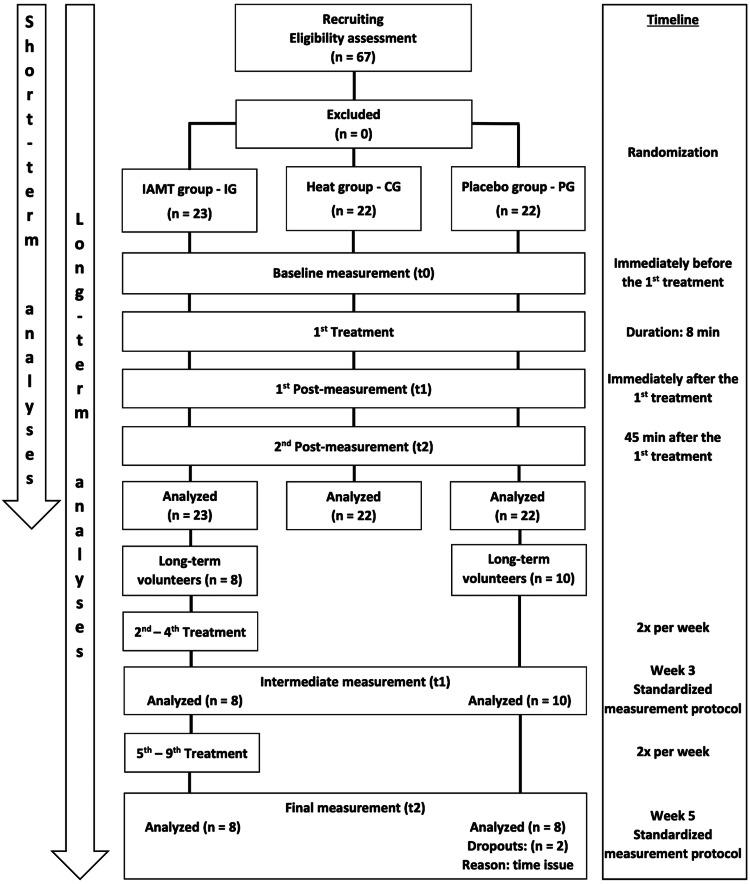
Flow chart of the short-term and long-term part of this pilot study IAMT: instrument-assisted manual therapy; IG: intervention group; CG: control group; PG: placebo group.

## Appendices

**Table 6:** List of inclusion and exclusion criteria

Inclusion criteria	Exclusion criteria
Female soccer player	Back pathology in the past 4 weeks
Age of 15–35 years	Chronic illnesses
Good health	Acute injuries
Active involvement in competition	
Minimum of three 90 min practice sessions per week	
